# Lipid Droplet Signaling in Metabolic Health and Aging

**DOI:** 10.1093/geroni/igab046.1761

**Published:** 2021-12-17

**Authors:** Charles Najt, Douglas Mashek

**Affiliations:** 1 University of Minnesota; 2 University of Minnesota, Minneapolis, Minnesota, United States

## Abstract

Lipid droplets (LDs) are neutral lipid rich organelles involved in lipid storage, fatty acid trafficking, and signaling. Emerging evidence from our laboratory and others suggests that the specific LD resident proteins couple/uncouple cells and tissues from inflammation and metabolic dysfunction. However, the mechanism by which LD proteins influences these critical pathways remains unknown. We will present data delving into the role of LD proteins Perilipin (PLIN) 2 and 5 in balancing cellular energy metabolism, mitochondrial function, and inflammation. Data will be presented defining novel mechanisms through which PLIN2 orchestrates eicosanoid production as a means to promote inflammation. We will contrast these findings to PLIN5, which uncouples LD accumulation from metabolic dysfunction and inflammation, in part due to its promotion of SIRT1 signaling. Overall, these studies will highlight a crucial role of LD metabolism and signaling in regulating cellular energy homeostatic processes known to be key players in governing healthspan.

